# Restriction of salt, alcohol and coffee intake and Ménière’s disease: insight from Mendelian randomization study

**DOI:** 10.3389/fnut.2024.1460864

**Published:** 2024-09-16

**Authors:** Wei Gao, Pengwei Ma, Zi Wang, Jianing Guo, Yuqiang Lun, Weilong Wang, Hao Yuan, Siyu Li, Rui Liang, Lianjun Lu

**Affiliations:** Department of Otolaryngology Head and Neck Surgery, Tangdu Hospital, The Fourth Military Medical University, Xi’an, China

**Keywords:** Ménière’s disease, dietary restriction, low-salt diet, alcohol consumption, coffee consumption, Mendelian randomization

## Abstract

**Background:**

Restricting salt, caffeine, and alcohol intake is commonly recommended as a first-line treatment for patients with Ménière’s disease (MD). However, it remains unclear whether these interventions effectively improve symptoms of MD. Therefore, we conducted a bidirectional two-sample Mendelian randomization (MR) analysis to evaluate the relationship between these dietary modifications and MD.

**Methods:**

Summary statistics for salt added to food, alcohol consumption, coffee consumption, and MD were sourced from the United Kingdom Biobank, GSCAN, and the FinnGen study, involving up to 941,280 participants. The main analyses were performed using the random-effects inverse-variance weighted (IVW) approach and were complemented by four additional methods. Multiple sensitivity analyses were performed to validate the findings, and both forward and reverse MR analyses were employed to address potential reverse causality bias.

**Results:**

The primary MR results using the IVW method revealed that salt added to food (OR = 0.719, 95% CI: 0.429–1.206; *p* = 0.211), alcohol consumption (OR = 0.834, 95% CI: 0.427–1.628; *p* = 0.595), and coffee consumption (OR = 0.852, 95% CI: 0.555–1.306; *p* = 0.461) were not significantly correlated with MD. In reverse analysis, no evidence of significant effect was found from MD to salt added to food (OR = 1.000, 95% CI: 0.993–1.007; *p* = 0.957), alcohol consumption (OR = 0.998, 95% CI: 0.987–1.008; *p* = 0.682), and coffee consumption (OR = 0.998, 95% CI: 0.985–1.011; *p* = 0.72).

**Conclusion:**

This MR analysis did not identify convincing evidence to support the idea that restricting salt, caffeine, and alcohol intake is beneficial for the treatment of MD.

## 1 Introduction

Ménière’s disease (MD) is a chronic inner ear disorder characterized by recurrent spontaneous vertigo lasting from 20 min to hours, fluctuating sensorineural hearing loss, aural fullness, and tinnitus (1, 2). It may affect one or both ears and commonly occurs between the ages of 40 and 60 years with a slight female preponderance. The estimated incidence is 190 per 100,000 people annually, based on a large study in the US (3). Although endolymphatic hydrops (EH) is the most consistent histopathological finding in patients with MD, its true mechanism remains poorly understood (4) and effective nonablative treatments remain elusive (5). However, there are many therapeutic options ranging from dietary changes, to medicines and in some cases surgery. Currently, dietary modifications, including restriction of salt, caffeine and alcohol intake, are widely recommended to MD patients as a first-line treatment or as a foundational management strategy in conjunction with other interventions (6–8).

Salt intake can affect the concentrations of electrolytes, potentially contributing to endolymphatic hydrops (EH). Therefore, restricting salt intake is believed to be helpful in lowering endolymphatic pressure and reducing the number of vertigo attacks (9–11). Additionally, caffeine and alcohol intake are thought to exacerbate symptoms in patients with MD by constricting of blood vessels of inner ear (10, 12). However, a comprehensive systematic review found no randomized controlled trial or quasi-randomized controlled trial investigating the role of dietary modifications, including restriction of salt, caffeine, and alcohol, in the treatment of MD (5).

Given the prevalence of processed foods and food additives in contemporary diets, conducting a randomized controlled trial (RCT) to investigate dietary modifications, especially those involving the restriction of salt, caffeine, and alcohol, poses significant challenges. Furthermore, Mendelian randomization (MR) offers a promising alternative, operating similarly to a quasi-randomized study, where genetic variations are randomly assorted during meiosis (13). By utilizing dietary-related genetic variations as instrumental variables (IVs) in MR analyses, the causal association between dietary modification and MD could be effectively assessed.

## 2 Materials and methods

### 2.1 Study design

**Figure 1:**
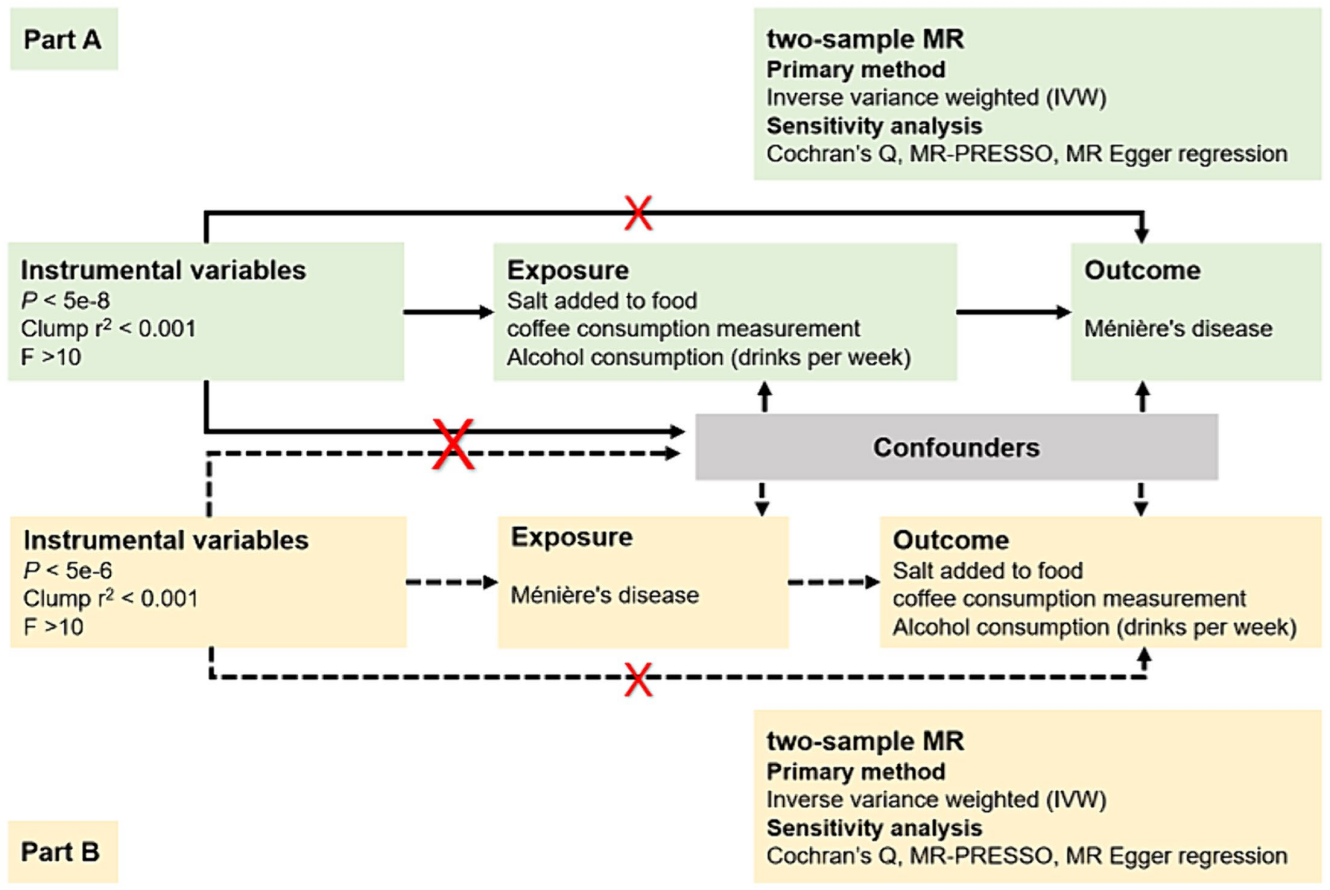
The overview design for MR study.

In response to the ongoing controversy regarding whether dietary modifications, such as restricting salt, caffeine, and alcohol intake, should be widely recommended as a first-line treatment for patients with MD (14), we conducted a bidirectional two-sample MR study in the European population. As illustrated in Figure 1, this study has two main components: an analysis of causal effects of three most debated dietary habits on MD (Part A) and an analysis of causal effects of MD on these dietary habits (Part B); Multiple single nucleotide polymorphisms (SNPs) representing genetic variation were selected as Instrumental variables (IVs), and they needed to meet the following requirements: (1) IVs are robustly associated with exposure data; (2) IVs are not associated with potential confounders; and (3) IVs only affect the outcome via the exposure (15).

This study was a secondary analysis of publicly available genome-wide association studies (GWAS) summary statistics, and no individual-level data was utilized. Ethical approval and informed consent could be obtained in the original GWAS studies. Therefore, no separate ethics statement was required for this study.

### 2.2 Data sources

According to previous studies, adding salt to cooked food has been identified as a predictor of dietary salt intake levels (16). Therefore, we used the IEU OpenGWAS project to obtain the GWAS dataset of salt added to food from United Kingdom Biobank, which is the largest GWAS comprising 462,630 European-ancestry individuals. Genetic data for coffee consumption was also acquired from the UK Biobank, including 448,204 participants of European ancestry (17). Additionally, summary data for alcohol consumption (drinks per week) was sourced from a genome-wide meta-analysis, GWAS & Sequencing Consortium of Alcohol and Nicotine use (GSCAN), which involved 941,280 participants of European ancestry (18).

To avoid bias caused by overlap, the GWAS data for MD were derived from the tenth version of the Finngen consortium,^1^ encompassing 395,179 participants (2,977 cases and 392,202 controls). Detailed information on these GWAS datasets was provided in Table 1.

**Table 1:** Information of GWAS data sources.

Data source	Trait	N case	Total individual	Ethnicity	PMID	GWAS id	Number of SNPs^**^	Year
UK biobank	Salt added to food	–	462,630	European	–	ukb-b-8121	9,851,867	2018
UK biobank	Coffee consumption measurement	–	448,204	European	32,193,382	GCST90132984	11,014,716	2020
GSCAN^*^	Alcohol consumption (drinks per week)	–	941,280	European	30,643,251	GCST007461	11,916,706	2019
FinnGen	Ménière’s disease	2,977	392,202	European	–	–	21,306,069	2023

*GSCAN: GWAS and Sequencing Consortium of Alcohol and Nicotine use. **SNPs: single nucleotide polymorphisms.

### 2.3 Selection of instrumental variables

To ensure that the IVs fulfilled the three fundamental assumptions of MR with sufficient validity, we implemented several measures to guarantee the quality of instrumental SNPs used in our study. First, we selected SNPs that reached genome-wide significance (*p* < 5 × 10^−8^), indicating a strong association with exposures. However, due to limited SNPs for MD in reverse MR analysis when selecting IVs with a *p* value <5 × 10^−8^, we relaxed the selection criteria (*p* < 5 × 10^−6^) to obtain more correlation results. Second, we conducted a linkage disequilibrium clumping to ensure the independence of all instrumental SNPs utilized in our study (window size = 10,000 kb, *r*^2^ < 0.001). Third, we calculated the F statistic for each SNP using the formula: F = *β*^2^/SE^2^, where β represents the estimated effect of the SNP on the exposure, and SE is the standard error of this estimate. SNPs with an F statistic less than 10 were removed, as they were considered weak instruments with low statistical power (19). Fourth, we performed a comprehensive look-up of all SNPs employed in this research in PhenoScanner V2 to investigate any pleiotropic associations with other phenotypes at the genome-wide significance level that may be associated with outcomes (20). Finally, palindrome SNPs were removed to prevent the influence of alleles on the causal relationship between dietary habits and MD. In cases where there was no SNP associated with exposure in the outcome GWAS, a proxy SNP significantly associated with the variation of interest (*r*^2^ > 0.8) was selected.

### 2.4 Statistical analyses

Based on the IVs selected above, we performed a two-sample MR analyses using R software (version 4.3.1, R Foundation for Statistical Computing, Vienna, Austria) and its associated R package, “TwoSampleMR” (version 0.5.7). Five different methods of MR analysis were used in our study. The inverse variance weighted (IVW) method served as primary approach to explore the possible causal association between dietary habits and MD. This method calculated the inverse-variance weighted mean of ratio estimates from two or more instruments and assumed that all SNPs used were valid. To address potential heterogeneity in our study, we utilized the random-effects IVW approach instead of the fixed IVW, which might produce overly precise results in the presence of heterogeneity. MR-Egger, weighted median, simple mode, and weighted mold were employed as Supplementary methods to further assess the causal association.

To ensure the reliability and robustness of the causality assessment results, several essential sensitivity analyses were conducted. Cochran’s Q test was employed to test for heterogeneity. MR-Pleiotropy Residual Sum and Outlier (MR-PRESSO) was used to detect and correct for horizontal pleiotropy by identifying and removing outlier genetic variants that could introduce bias in the IVW estimate. The MR Egger regression intercept was utilized to estimate potential pleiotropy of SNP, with a *p*-value >0.05 indicating no horizontal pleiotropy. A leave-one-out analysis was also conducted to detect and verify the presence of unusual instrumental variables that significantly affected the estimation of causal effects. Subsequently, a reverse MR analysis was performed to examine whether a reverse causal association existed between MD and dietary habits. Results were presented as odds ratios (OR) with respective 95% confidence intervals (CI). *p*-values were two-sided and statistical significance was set at the 0.05.

## 3 Results

### 3.1 Salt added to food and MD

**Figure 2:**
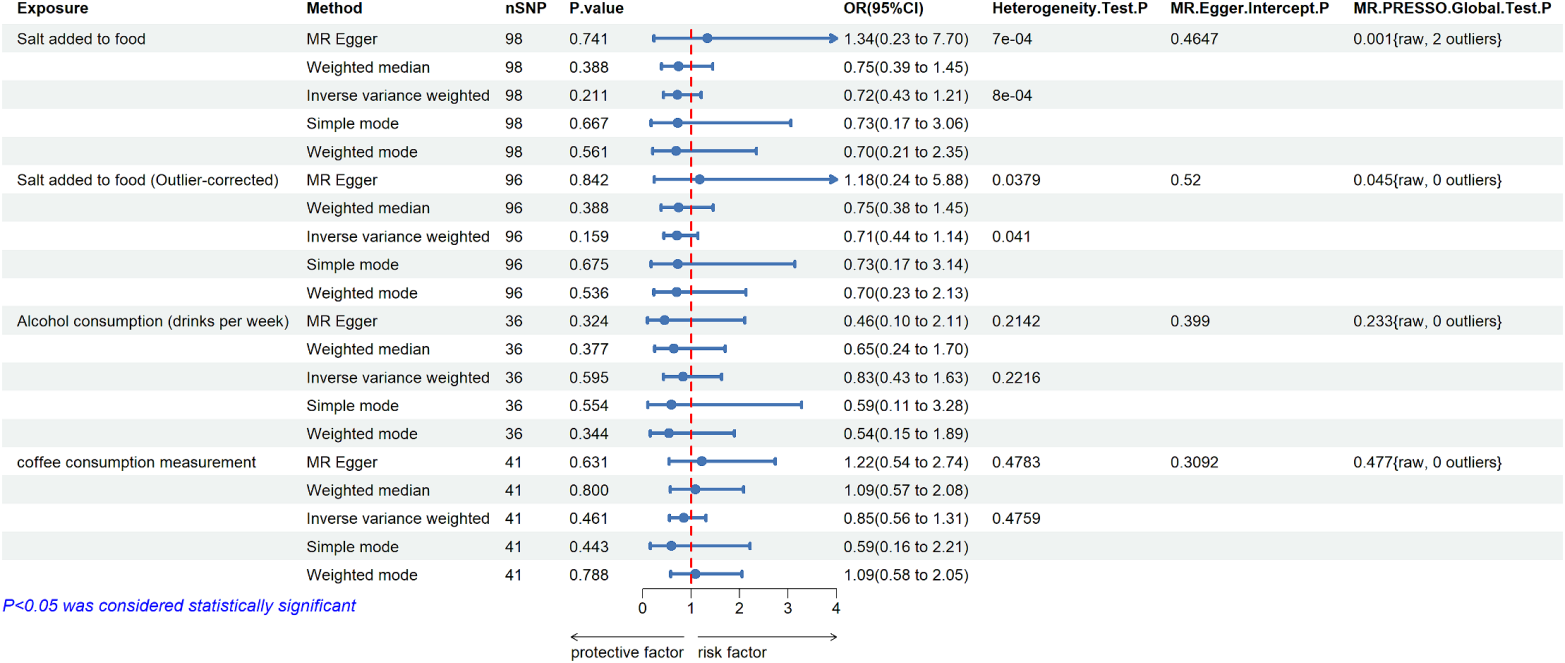
MR estimates the casual effect of Salt added to food, Alcohol consumption and Coffee consumption measurement on MD (nSNP: number of single nucleotide polymorphisms; OR: odds ratios; 95% CI: 95% confidence intervals; MR-Egger: mendelian randomization Egger regression; MR-PRESSO: mendelian randomization pleiotropy residual sum and outlier).

After several screenings, 98 SNPs were finally utilized as IVs for salt added to food, with basic information provided in Supplementary Table S1. The distribution of F-statistics corresponding to single SNPs ranged from 29.74 to 224.90 (median 39.48 (IQR 32.12–50.90)), indicating that causal associations were less likely to be affected by weak instrumental variables. The IVW method suggested no significant association between salt added to food and MD (OR = 0.719, 95% CI: 0.429–1.206; *p* = 0.211). Although heterogeneity among SNPs was detected in Cochran’s Q test by both MR-Egger regression and IVW method, the results of the MR-Egger regression (OR = 1.343, 95% CI: 0.234–7.702; *p* = 0.741), simple model (OR = 0.729, 95% CI: 0.174–3.058; *p* = 0.667), weighted model (OR = 0.697, 95% CI: 0.207–2.349; *p* = 0.561) and especially weighted median (OR = 0.748, 95% CI: 0.387–1.447; *p* = 0.388) also indicated no directional causality effect (Figure 2; Supplementary Table S5; Supplementary Figure S1). Additionally, the difference between the MR-Egger regression intercept term and 0 was not statistically significant (*p* > 0.05), indicating no genetic pleiotropy among the SNPs (Supplementary Table S6). However, the MR-PRESSO method identified two outlier SNPs (rs7465705 and rs6987313); Nevertheless, even after excluding these outliers, the main analysis results remained insignificant (outlier-corrected IVW: OR = 0.711, 95% CI: 0.442–1.144; *p* = 0.159).

**Figure 3:**
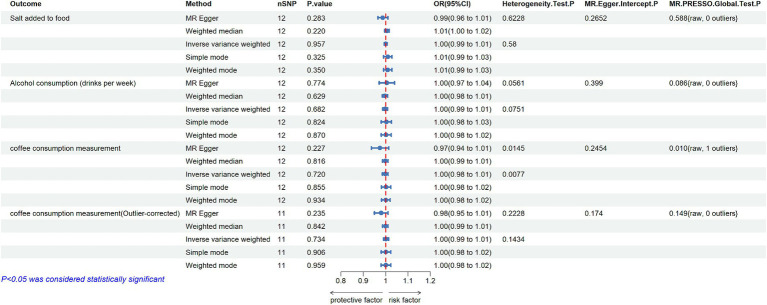
MR estimates the casual effect of MD on Salt added to food, Alcohol consumption and Coffee consumption measurement (nSNP: number of single nucleotide polymorphisms; OR: odds ratios; 95% CI: 95% confidence intervals; MR-Egger: mendelian randomization Egger regression; MR-PRESSO: mendelian randomization pleiotropy residual sum and outlier).

In the reverse MR study, 12 SNPs were identified as IVs for MD after quality control (Supplementary Table S2). The primary results were presented in Figure 3. The IVW method indicated that there was no significant evidence to support a causal effect from MD to salt added to food (OR = 1.000, 95% CI: 0.993–1.007; *p* = 0.957). Furthermore, the statistic Q shown by MR-Egger regression and Cochran Q test of IVW method, both with *p* > 0.05, suggested that there was no heterogeneity among SNPs. Based on the MR-PRESSO analysis, no potential outlier was detected (*p* = 0.588), and the use of the MR-Egger intercept method did not show any substantial directional pleiotropy among the selected IVs (*p* = 0.265). Moreover, no conflicting result was found by other MR methods (Supplementary Tables S7, S8), and the result of the leave-one-out sensitivity analysis demonstrated the stability of the results (Supplementary Figure S2).

### 3.2 Alcohol consumption and MD

The analysis of genetically predicted alcohol consumption (drinks per week) on MD yielded 36 SNPs, with F-statistics ranging from 28.38 to 964.41 (Supplementary Table S3). The IVW method revealed no significant casual association between alcohol consumption and MD (OR = 0.834, 95% CI: 0.427–1.628; *p* = 0.595). Furthermore, the results of the MR-Egger regression (OR = 0.459, 95% CI: 0.100–2.111; *p* = 0.324), weighted median (OR = 0.646, 95% CI: 0.245–1.703; *p* = 0.377), simple model (OR = 0.594, 95% CI: 0.107–3.282, *p* = 0.554) and weighted model (OR = 0.542, 95% CI: 0.155–1.893; *p* = 0.344) also demonstrated consistent insignificant effects of alcohol consumption on MD (Figure 2; Supplementary Table S5; Supplementary Figure S1). Additionally, the findings of Cochran’s Q test, which incorporated both the IVW and MR-Egger methods, revealed no significant heterogeneity among the selected IVs (Supplementary Table S6). Besides, the horizontal pleiotropy was not detected by MR-Egger regression intercept (*p* > 0.05), and no outlier SNPs were found by using the MR-PRESSO test. Finally, leave one out analysis showed that the results remained robust after removing any one of the SNPs (Supplementary Figure S1).

In reverse MR study, the same 12 SNPs mentioned above were used as IVs for MD. All estimated results of different methods were displayed in Figure 3. The IVW method showed that there was no evidence to support a positive or negative casual effect from MD to alcohol consumption (OR = 0.998, 95%CI: 0.987–1.008; *p* = 0.682). The MR-Egger regression method yielded a similar result (OR = 1.005 95%CI: 0.971–1.040; *p* = 0.774), as well as the weighted median method (OR = 0.997, 95%CI: 0.985–1.009; *p* = 0.629). The Cochran’s Q test indicated no evidence of heterogeneity (*p* > 0.05), and no outlier was identified in the MR-PRESSO model (Figure 3; Supplementary Tables S7, S8; Supplementary Figure S2).

### 3.3 Coffee consumption and MD

In this analysis, 41 SNPs were finally included to estimate the causal effect from coffee consumption to MD. The F-statistics for every instrument were greater than 10 (from 28.37 to 829.69), indicating that these SNPs were strong instruments (Supplementary Table S4). The IVW method showed that the genetic tendency to coffee consumption was not associated with an increased risk of MD (OR = 0.852, 95%CI: 0.555–1.306; *p* = 0.461). Other MR methods yielded consistent results (Figure 2; Supplementary Table S5; Supplementary Figure S1). Additionally, the MR PRESSO Global test, MR Egger test, and Cochran’s Q test found no evidence of horizontal pleiotropy or heterogeneity across SNPs (Supplementary Table S6). Moreover, the *p* value for the intercept in MR-Egger was above 0.05, further indicating no evidence of pleiotropic effect.

In the reverse MR study, 12 eligible SNPs from MD GWAS data were used as IVs, and the results were shown in Figure 3. IVW analysis showed no association of genetically predicted MD with coffee consumption (OR = 0.998, 95%CI: 0.985–1.011, *p* = 0.72). Additionally, the results of other four MR methods showed a similar effect. Although the directional pleiotropy of MR-Egger did not show a significant difference, one outlier (rs72762919) was identified in the MR-PRESSO Global test. Heterogeneity was also found by Cochran’s Q test in both IVW and MR-Egger methods (*p* < 0.05). However, results did not change substantially after the removal of the outlier, and the Cochran’s Q test found no evidence of heterogeneity across SNPs when excluding the outlier (Figure 3; Supplementary Tables S7, S8; Supplementary Figure S2).

## 4 Discussion

This study represents the first attempt to utilize GWAS datasets to assess the potential causal impact of dietary salt, caffeine, and alcohol intake on MD using a bidirectional MR analysis. Our findings indicate that salt added to food, alcohol consumption and coffee consumption were not significantly associated with MD. Similarly, reverse direction analyses provided consistent evidence that genetic susceptibility to MD was not associated with a higher tendency toward these dietary habits. In other words, our study suggests that restricting salt, caffeine, and alcohol intake may not be beneficial for the treatment of MD.

The use of a low-salt diet as a treatment for MD has a longstanding history. The observation that water retention can exacerbate the symptoms of MD was first documented in 1929, leading to the proposal of reducing salt intake and fluid consumption as a potential treatment strategy (21). Subsequent uncontrolled observational studies reported improvements in MD symptoms with salt restriction, which have been further supported by recent investigations, often in conjunction with diuretic therapy (9, 22, 23). Mori and Miyashita proposed an alternative hypothesis suggesting that MD symptoms worsen due to low levels of aldosterone, implying that a low-salt diet could alleviate symptoms by increasing aldosterone activity through the renin–angiotensin–aldosterone system (24, 25). However, the scientific support for salt restriction in managing MD remains limited, primarily due to the absence of high-quality evidence. A Cochrane systematic review, for instance, failed to identify studies meeting rigorous inclusion criteria, precluding definitive conclusions regarding the efficacy of salt restriction (5, 14). In our study, we employed MR analysis, a type of quasi-randomized study, in a large population to address this question, ultimately finding that salt restriction was ineffective in managing MD.

While alcohol consumption has been implicated as a possible factor in inner ear disorders, including MD, the evidence regarding its effects remains inconclusive (9, 12, 14). Despite common recommendations for MD patients to limit alcohol intake, no RCTs have been published to support this practice, as noted in a Cochrane review (5). However, observational studies have reported conflicting findings. For example, one study involving 72 patients with MD suggested that alcohol consumption may delay the onset of MD (26), while a large cohort study in Korea suggested alcohol consumption as a protective factor for adult men with MD (27). Nevertheless, it’s important to note that these studies may have overlooked potential confounders and failed to consider the toxic effects of alcohol. Therefore, alcohol consumption could not be considered as a treatment for MD. In our study, we found no correlation between alcohol consumption and MD, neither positively nor negatively, contributing further evidence to this complex issue.

Dietary coffee restriction has been proposed as a management strategy for MD, hypothesized to be effective due to the potential impact of caffeine on endolymph volume through sympathomimetic and diuretic actions (9, 12). Despite its routine recommendation for MD patients experiencing vertigo, the evidence supporting this approach is limited. Previous questionnaire investigations have suggested that a caffeine-free diet may be associated with a reduction in vertigo symptoms and an improvement in the functional level of the disease, as rated using the AAO-HNS scale (22). Additionally, a recent observational case–control study found that MD patients reported higher daily caffeine intake compared to unaffected individuals (28). However, our study, characterized by a larger population size and a quasi-randomized design, found no association between coffee consumption and the risk of MD. These findings contribute valuable insights to the existing literature on dietary interventions for MD management.

Taken in together, it is necessary to reconsider the value of restricting salt, caffeine, and alcohol intake for the treatment of MD. In light of our study, no significant correlation was found between these dietary modifications and MD. Relying solely on these restrictions may, to some extent, delay the use of more appropriate and effective treatments and could also have potentially negative implications for social, work, and family life (14). Therefore, the first advantage of this study was eliminating the controversy of dietary modification efficacy in MD treatment. Secondly, compared to observational clinical studies, MR is a more cost-effective, quicker, and more reliable method to evaluate the effects of interventions on MD. Thirdly, a series of sensitivity analyses were conducted to validate the robustness of our MR results. However, limitations should be considered when interpreting the results of this study. Since all the participants included in our study were restricted to European ancestry, the results may be biased and may not apply to other races. In the absence of individual data, it was not possible to conduct stratified analyses of MD subtypes and severity. Moreover, the limited SNPs for MD may lead to some bias in the results of reversed analysis, and MD is a multifactorial disease with more than one etiology converging into the characteristic symptomatology, making it challenging to identify confounding factors that could introduce bias into the results. Finally, the causal effect of genetic variants exposure on the outcome can be modified by compensatory processes during development, therefore the results of this study must be viewed with caution in future analyses.

## 5 Conclusion

In summary, this MR study did not find convincing evidence to support the idea that restricting salt, caffeine, and alcohol intake is beneficial for the treatment of MD. These findings offer new insights into the role of dietary modification in MD and suggest that further confirmation through appropriately designed RCT is needed.

## Data Availability

The original contributions presented in the study are included in the article/Supplementary material, further inquiries can be directed to the corresponding author.

## Footnotes

^1^ https://r10.finngen.fi/

## Supplementary material

Supplementary Table S1 The Salt added to food-related genetic variants used for the MR analyses.

Supplementary Table S2 The Ménière's disease-related genetic variants used for the reverse MR analyses.

Supplementary Table S3 The Alcohol consumption (drinks per week)-related genetic variants used for the MR analyses.

Supplementary Table S4 The Coffee consumption measurement-related genetic variants used for the MR analyses.

Supplementary Table S5 The causal effects of Salt added to food, Alcohol consumption and Coffee consumption measurement on MD.

Supplementary Table S6 Mendelian randomization Sensitivity analysis of Salt added to food, Alcohol consumption and Coffee consumption measurement on MD.

Supplementary Table S7 The causal effects of MD on Salt added to food, Alcohol consumption and Coffee consumption measurement.

Supplementary Table S8 Mendelian randomization Sensitivity analysis of MD on Salt added to food, Alcohol consumption and Coffee consumption measurement.
