# Supplementary material for: Restriction of salt, alcohol and coffee intake and Ménière’s disease: insight from Mendelian randomization study
Source: Front Nutr. 2024 Sep 16;11:1460864. doi: 10.3389/fnut.2024.1460864 (PMC11439828; doi:10.3389/fnut.2024.1460864)
Supplement: Supplementary file 2 [file Image_1.pdf]

Supplementary Material

1 Supplementary Figures

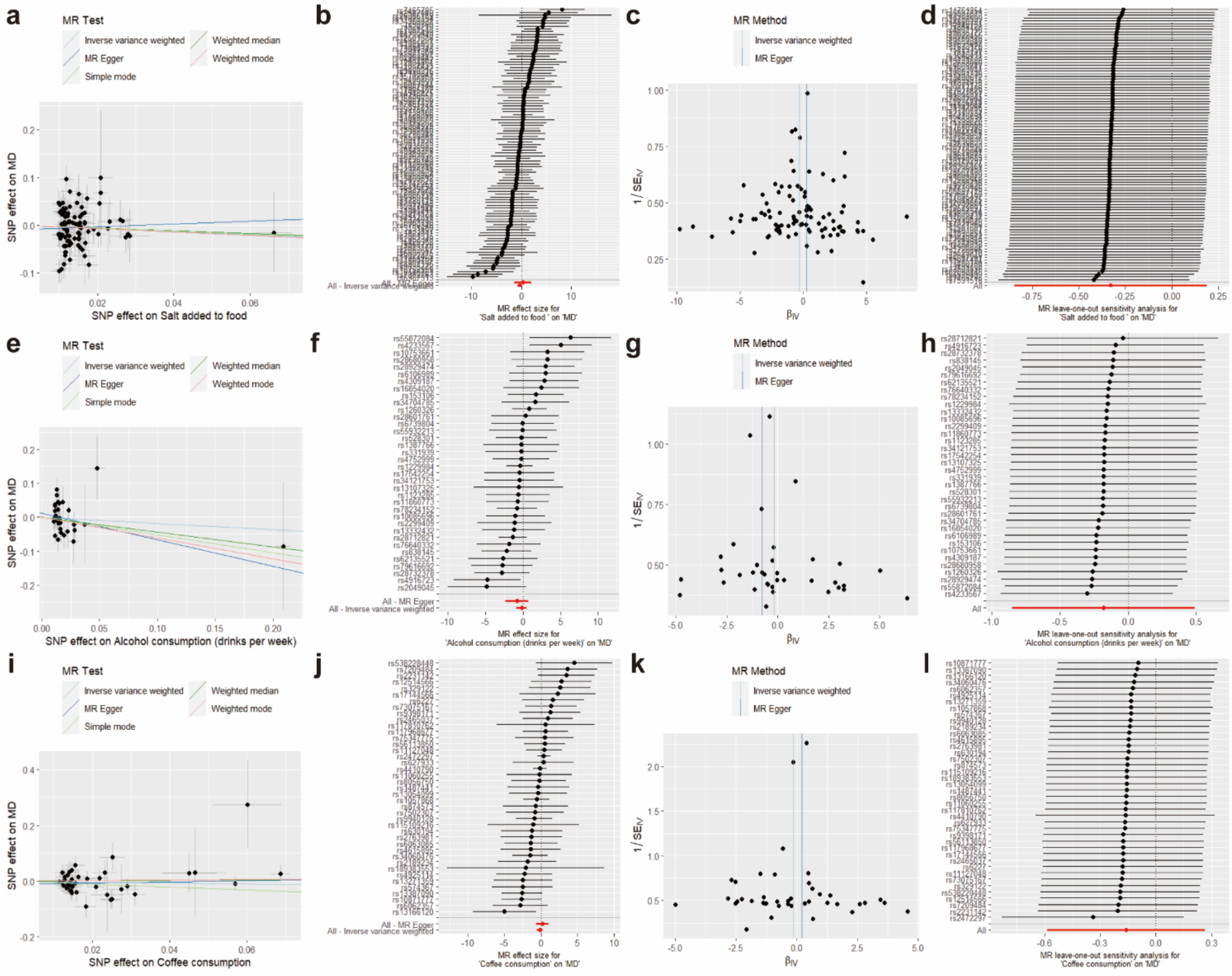

Supplementary Figure S1. Associations between single-nucleotide polymorphism instruments for Salt added to food(a - d), Alcohol consumption(e - h), Coffee consumption measurement(i - l)

(exposures) and Meniere's Disease (outcome): (a, e, i) Scatterplots, (b, f, j) Forest plots, (c, g, k) Funnel plots, (d, h, l) Leave-one-out plots.

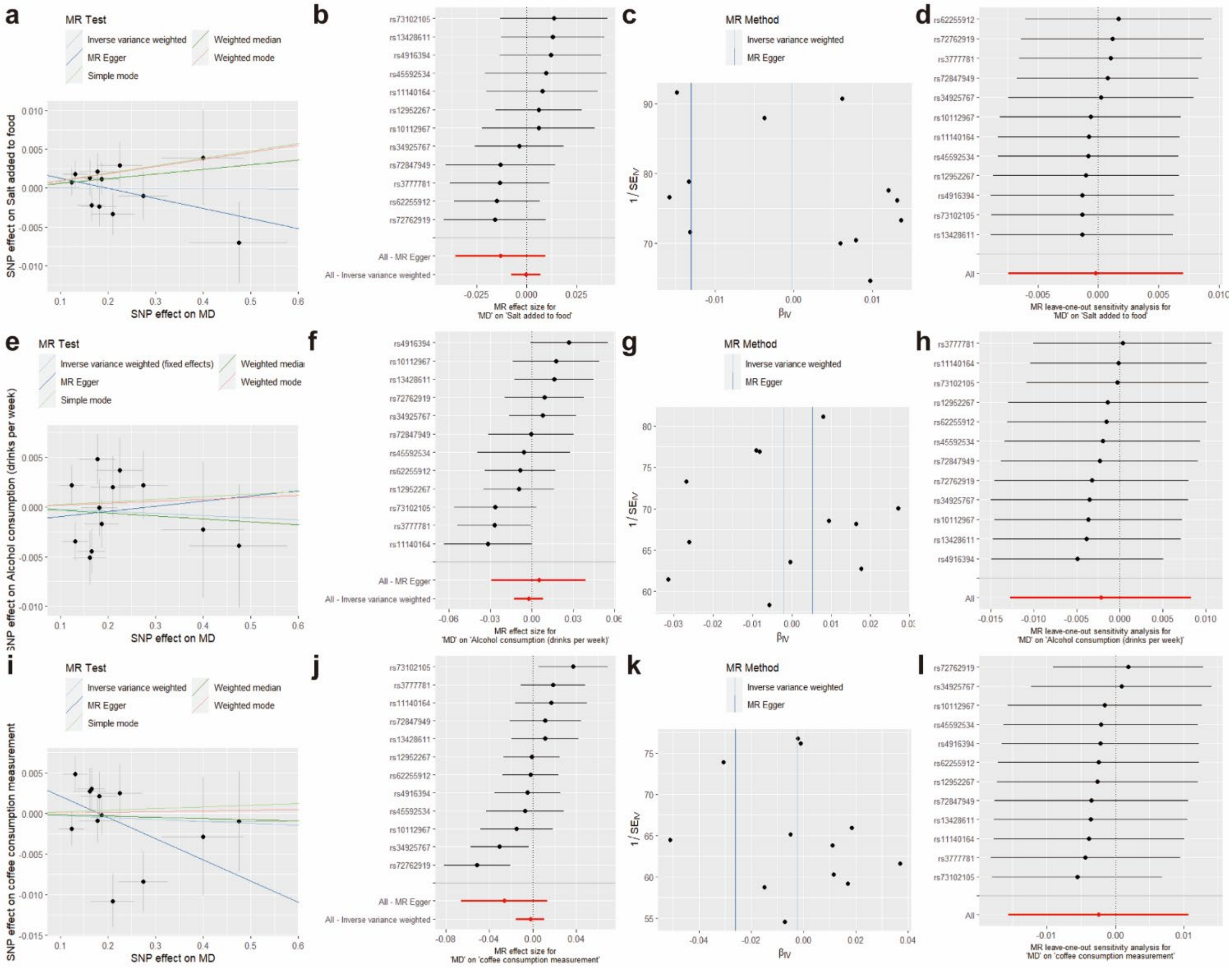

Supplemental Figure S2. Associations between single-nucleotide polymorphism instruments for Meniere's Disease (exposure) and Salt added to food(a – d), Alcohol consumption(e – h), Coffee consumption measurement(I - l) (outcomes): (a, e, i) Scatterplots, (b, f, j) Forest plots, (c, g, k) Funnel plots, (d, h, l) Leave-one-out plots.
