# Supplementary material for: Restriction of salt, alcohol and coffee intake and Ménière’s disease: insight from Mendelian randomization study
Source: Front Nutr. 2024 Sep 16;11:1460864. doi: 10.3389/fnut.2024.1460864 (PMC11439828; doi:10.3389/fnut.2024.1460864)
Supplement: Supplementary file 3 [file Table_2.DOCX]

Supplementary Material

# Supplementary Tables

Table S1 The Salt added to food-related genetic variants used for the MR analyses.

Table S2 The Ménière's disease-related genetic variants used for the reverse MR analyses.

Table S3 The Alcohol consumption (drinks per week)-related genetic variants used for the MR analyses.

Table S4 The Coffee consumption measurement-related genetic variants used for the MR analyses.

Table S5 The causal effects of Salt added to food, Alcohol consumption and Coffee consumption measurement on MD.

Table S6 Mendelian randomization Sensitivity analysis of Salt added to food, Alcohol consumption and Coffee consumption measurement on MD.

Table S7 The causal effects of MD on Salt added to food, Alcohol consumption and Coffee consumption measurement.

Table S8 Mendelian randomization Sensitivity analysis of MD on Salt added to food, Alcohol consumption and Coffee consumption measurement.

# Supplementary Figures


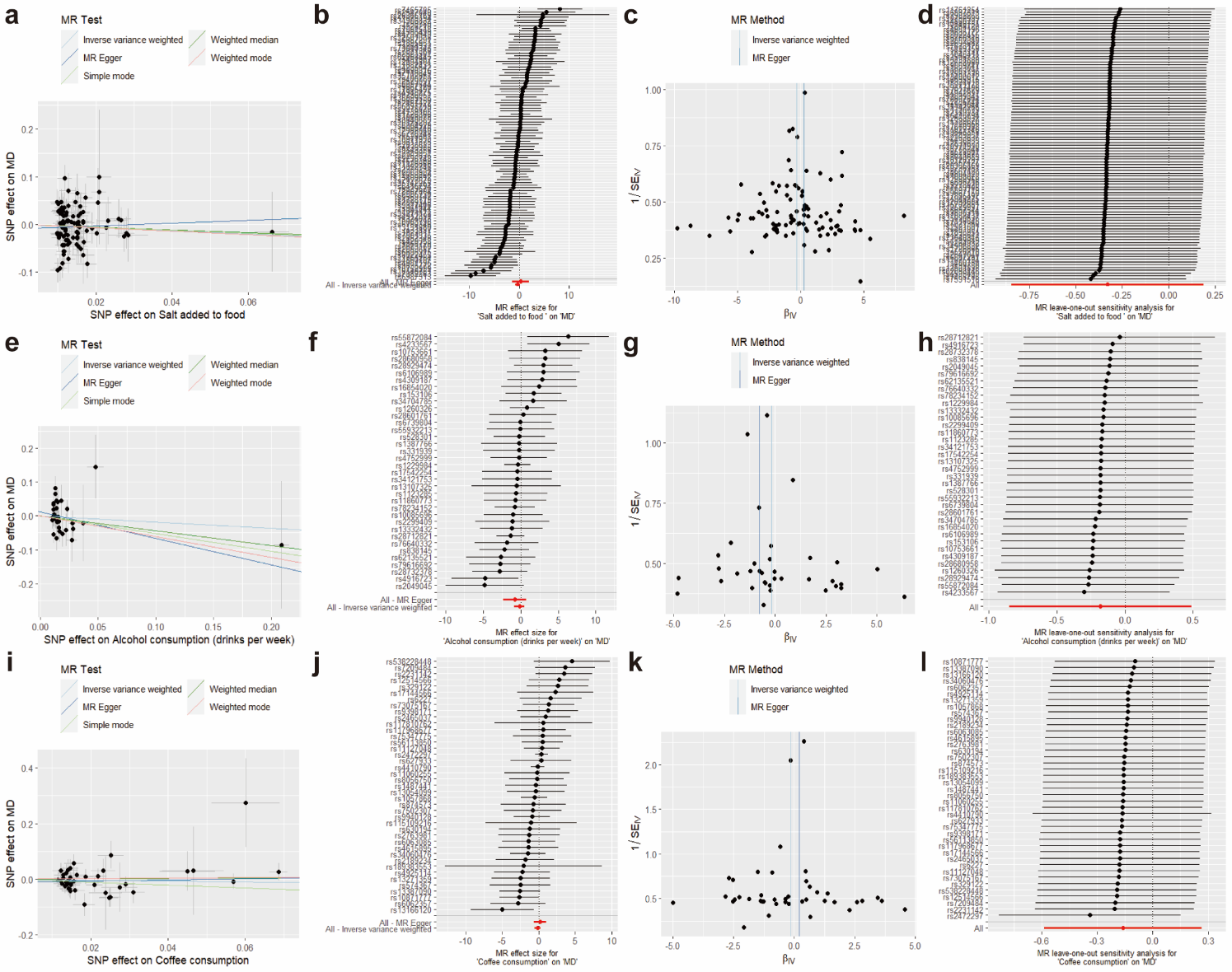


**Supplementary Figure S1.** Associations between single-nucleotide polymorphism instruments for Salt added to food(a - d), Alcohol consumption(e - h), Coffee consumption measurement(i - l) (exposures) and Meniere’s Disease (outcome): (a, e, i) Scatterplots, (b, f, j) Forest plots, (c, g, k) Funnel plots, (d, h, l) Leave-one-out plots.


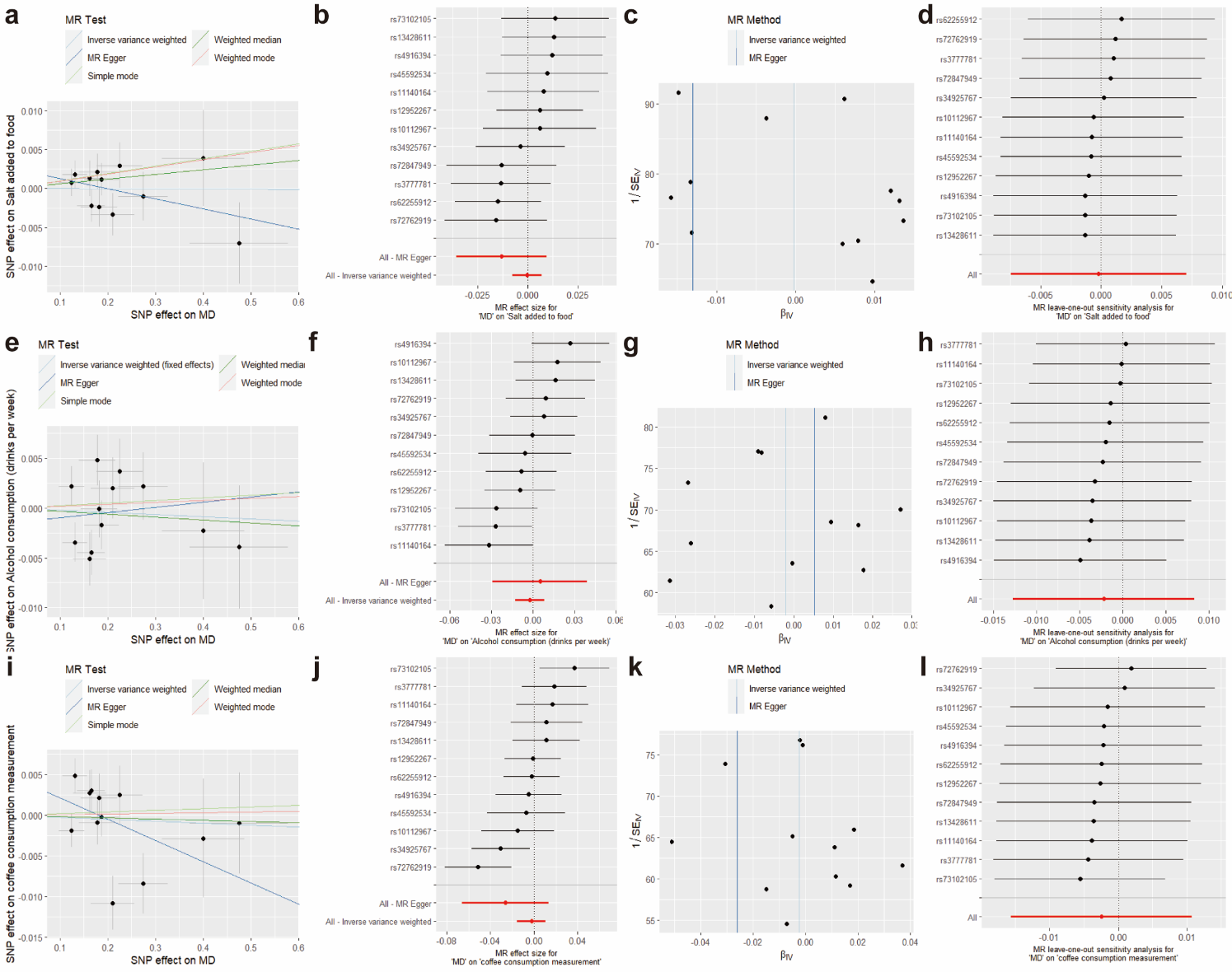


**Supplemental Figure S2.** Associations between single-nucleotide polymorphism instruments for Meniere’s Disease (exposure) and Salt added to food(a – d), Alcohol consumption(e – h), Coffee consumption measurement(I - l) (outcomes): (a, e, i) Scatterplots, (b, f, j) Forest plots, (c, g, k) Funnel plots, (d, h, l) Leave-one-out plots.
